# Structuring of plant communities across agricultural landscape mosaics: the importance of connectivity and the scale of effect

**DOI:** 10.1186/s12862-021-01903-9

**Published:** 2021-09-09

**Authors:** Michael McLeish, Adrián Peláez, Israel Pagán, Rosario Gavilán, Aurora Fraile, Fernando García-Arenal

**Affiliations:** 1 Centro de Biotecnología y Genómica de Plantas (CBGP, UPM-INIA), Universidad Politécnica de Madrid (UPM) – Instituto Nacional de Investigación y Tecnología Agraria y Alimentaria (INIA), and E.T.S.I. Agronómica, Alimentaria y de Biosistemas, Campus de Montegancedo, UPM, Pozuelo de Alarcón, 28223 Madrid, Spain; 2 Unidad de Botánica, Departamento de Farmacología, Farmacognosia y Botánica, Facultad de Farmacia, Universidad Complutense, Madrid, Spain

**Keywords:** Spatial autocorrelation, Scale of effect, Environmental heterogeneity, Movement ecology

## Abstract

**Background:**

Plant communities of fragmented agricultural landscapes, are subject to patch isolation and scale-dependent effects. Variation in configuration, composition, and distance from one another affect biological processes of disturbance, productivity, and the movement ecology of species. However, connectivity and spatial structuring among these diverse communities are rarely considered together in the investigation of biological processes. Spatially optimised predictor variables that are based on informed measures of connectivity among communities, offer a solution to untangling multiple processes that drive biodiversity.

**Results:**

To address the gap between theory and practice, a novel spatial optimisation method that incorporates hypotheses of community connectivity, was used to estimate the scale of effect of biotic and abiotic factors that distinguish plant communities. We tested: (1) whether different hypotheses of connectivity among sites was important to measuring diversity and environmental variation among plant communities; and (2) whether spatially optimised variables of species relative abundance and the abiotic environment among communities were consistent with diversity parameters in distinguishing four habitat types; namely Crop, Edge, Oak, and Wasteland. The global estimates of spatial autocorrelation, which did not consider environmental variation among sites, indicated significant positive autocorrelation under four hypotheses of landscape connectivity. The spatially optimised approach indicated significant positive and negative autocorrelation of species relative abundance at fine and broad scales, which depended on the measure of connectivity and environmental variation among sites.

**Conclusions:**

These findings showed that variation in community diversity parameters does not necessarily correspond to underlying spatial structuring of species relative abundance. The technique used to generate spatially-optimised predictors is extendible to incorporate multiple variables of interest along with a priori hypotheses of landscape connectivity. Spatially-optimised variables with appropriate definitions of connectivity might be better than diversity parameters in explaining functional differences among communities.

**Supplementary Information:**

The online version contains supplementary material available at 10.1186/s12862-021-01903-9.

## Background

Landscape connectivity either facilitates or impedes the movement of species among resources of an ecosystem [1]. In agricultural ecosystems the spatial arrangement and connectivity of wild and anthropic plant communities (i.e., their configuration) influences biodiversity as well as their functional diversity [2, 3]. Functional diversity describes differences among species and the functional roles they perform in ecosystems. However, landscape-scale studies typically characterise compositional differences among communities in terms of species richness and abundance to demonstrate structure-function relationships, which may not correspond to biological processes [4–6]. Compositional variation of traits among communities, rather than species diversity per se, is expected to influence biological processes [7]. Instead of species diversity, spatially optimised predictors based on species compositional variation, have the potential to reveal biological processes even in the absence of information on traits.

As environmental variation may influence biological processes at multiple scales, it is difficult to couple processes with patterns of biodiversity [8–10]. As in all landscapes, agricultural ecosystems include habitat types that are connected physically and historically. For instance, climate and/or historical factors, and species invasions are associated with the distribution of species over large and small spatial scales, while local scale patterns tend to be driven by species interactions [11–14]. Spatial autocorrelation of species relative abundances is widely used for adjusting analyses to meet assumptions of dependence among communities [15]. However, spatial autocorrelation among experimental sites poses two main challenges to using community diversity as a predictor of biological processes [16, 17]. First, connectivity among study sites has to be specified, and second, the predictor variables selected for describing community characteristics have to capture the scale of effect of the processes being measured. Species interactions such as competition, parasitism, and mutualism, may be contingent on climate [18], the topology of a study area [19], the movement ecology of species among suitable patches [20], or landscape disturbance [21]. To untangle the effects of multiple processes on biodiversity, spatial structuring of species relative abundances and variation of the abiotic environment among both isolated and well-connected plant communities need to be considered.

The term ‘connectivity’ has been used in the field of landscape ecology to describe the movement of an organism through a landscape as a function of distance and landscape structure [1, 22, 23]. Connectivity is an important concept in community ecology because it affects dispersal among local communities and species interactions within them [24–26], and sustains ecosystem function [27, 28]. For example, ecological networks have shown that a majority of studies that use weighted distance measures between agents of interest (e.g., species, communities) may not make sense when interpreting biological processes such as the probability of dispersal, interaction frequency, contact rate, or carbon flow [29]. Studies that exploit differences in space as experimental conditions, select study sites (or plots) that are generally not contiguous, with intervening patches and variation in the amount of habitat surrounding each site. For example, there was a significant positive effect between the proportion of crop land compared to unmanaged land, and both plant virus prevalence and aphid vector community richness [30]. In another study, soil management practices were found to correspond to variation in predator community abundance, ground-dwelling arthropods, and aphid predation [31]. Traditional approaches often assume that the matrix surrounding patches of interest is uniform [32]. However, variation in the type of interpatch matrix and the connectivity among study sites are expected to contribute significantly to patch isolation and affect local assembly and the stability of communities.

One solution to correcting for spatial biases is to optimise predictor variables by removing the variation within covariates as explained by connectedness. Spatially optimised predictor variables (i.e., with the scale of effect identified) may be more strongly related to the effect of the process being investigated, compared to the observed measurements from which the optimised variables are derived (e.g., species relative abundance). For example, one multi-scale approach is to select a number of scales of effect for each predictor based on known biological gradients [33]. However, high species density variance over space in landscapes comprising both wild and managed communities, makes the identification of scales of effect based on correlations between ecological factors at increasing distances from focal sites, impractical in most instances [8]. In highly mosaicked landscapes, spatial optimisation techniques that do not rely on a priori information about biological gradients may be a better approach. For example, to separate the effects of environmental filtering from diversity patterns, a partial Mantel test was used to show that environmental effects occurred largely independent of spatial effects on diversity among forest plots [34]. However, to validate the use of spatial optimisation approaches, hypotheses for the connectivity among the relative locations of species assemblages have to be determined a priori [35]. One way of achieving this is to compare hypotheses of connectivity that describe different densities of linear connections between study sites.

The aim of this study is to use a scale-optimisation approach in concert with hypotheses of connectivity [36] to investigate the variation among communities of multiple study sites of each of four habitat types. We expect diversity (i.e., abundance, richness, and evenness) estimators of sites of each habitat to be quantitatively similar. However, as the study sites are largely not spatially contiguous, and with some that adjoin each other, spatial structuring will depend on connectivity among study sites and environmental variability across the study area. To attain this aim, we detect scales of effect from empirical observations of plant species relative abundance and abiotic environmental variation. Four hypotheses of connectivity among the sites were described a priori to test the hypotheses that: (1) the scale of effect of differences in species diversity among the habitats depends on how connectivity among study sites is described; and (2) the diversity estimators used to characterise functional properties of communities are subject to spatial structuring and abiotic environmental variation. The resulting spatially optimised variables of the species relative abundances and the environmental factors may then be deployed in subsequent studies to test hypotheses of biological processes.

## Methods

### Study area and sampling

**Fig. 1:**
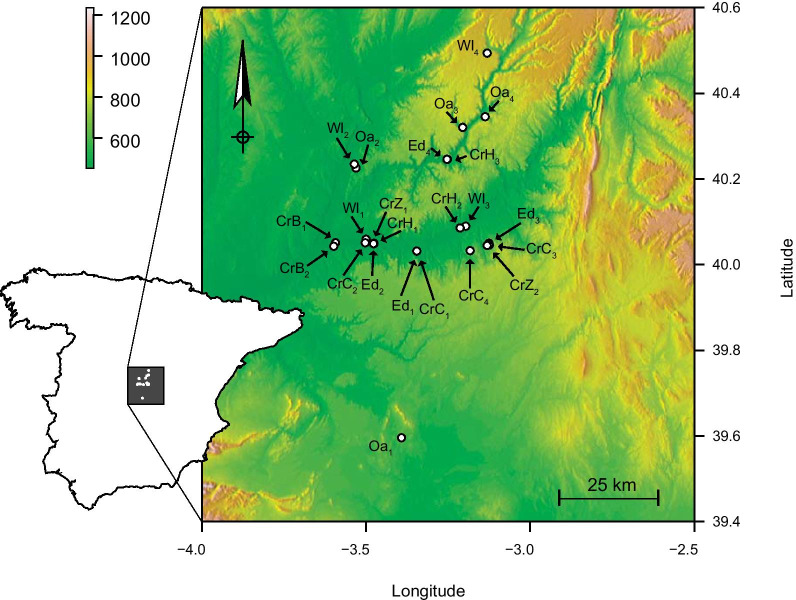
Extent of study area in central Spain. Sites indicated with open circles. The digital terrain map has a scale indicating elevation in metres (top left). Site code labels are Crop (Cr), Edge (Ed), Oak (Oa), and Wasteland (Wl); Crop codes are *Brassica* (B), *Hordeum* (H), *Cucumis* (C), and *Zea* (Z)

We performed this study between July 2015 and June 2017 in the Vega del Tajo-Tajuña agricultural region of the Tagus River Basin, in the South-Central Plateau of the Iberian Peninsula (Fig. 1). We conducted 78 individual collections that included 23 sampling sites, which comprised 329 plant species distributed over communities of four habitats with distinct cover types. The four habitat categories were nominated a priori to represent dominant land-cover types in the ecosystem and were distinguished by expert knowledge of community composition gained over twenty years of research in the region [24, 37–40]. We chose plant communities at sites present in forest (Oak), successional scrubland (Wasteland), and at the borders (Edge) between crops (Crop) to represent habitat categories (For details see: Additional file 1: Appendix S1).

Four sites each of Oak (*n* = 4 sites × 4 re-samples each) and Wasteland (*n* = 4 sites × 4 re-samples each) were visited with collections made in autumn and spring over two growing seasons. Edge (*n* = 2 sites × 6 re-samples + 2 sites × 5 re-samples) and Crop (*n* = 7 sites × 2 re-samples + 3 sites × 3 re-samples + 1 site × 1 re-sample) with four and eleven sites respectively, were visited in spring, summer, and autumn. Eleven sites were chosen to better characterise the variation expected from the Crop communities as cultivated fields are subject to crop rotation and fallow periods. Oak sites supported expansive assemblies and required a relatively large sample size to account for patch heterogeneity and rare (low frequency) species. Similarly, Wasteland sites were also subject to patch heterogeneity but in a smaller area than Oak (See Additional file 1: Appendix S1 for rationale of sampling effort). Crop collections comprised 4 fields of *Cucumis melo* (melon), 2 of *Zea mays* (maize), 2 of *Brassica oleracea* (cabbage and cauliflower), and 3 of *Hordeum vulgare* (barley), i.e., the major summer or winter crops in the area. In Oak and Wasteland sites, 25 × 25 m quadrats were marked out and 150 samples per site systematically collected at each resampling. In Edge and Crop, 50 samples from a 25 × 2 m area at each site were collected at each resampling. A boustrophedonic transect method (a line taken alternately from right to left and from left to right, and so on) was used in all instances except for Edge that have highly linear configurations. Depending on the habit of the species, a number of leaves from different parts of the individual were collected, each collection of leaves from the individual representing a single sample. The samples were harvested at fixed points along the transect. Individuals of each plant species were preserved at each collection and specimens assigned a provisional species, genus, or family rank prior to consultation with an herbarium for taxonomic assignments. The identifications were undertaken by Dr. Rosario Gavilán [41, 42]. The voucher specimens are available on request with permission from the authors.

### Habitat diversity

As incomplete sampling and unequal sample sizes were part of our sampling strategy, we assessed whether our estimates were near to those expected from complete collections using rarefaction of species richness. We used detrended correspondence analysis (DCA) conducted with the R (version 3.5.2) package [43] *vegan* [44] to visualise the homogeneity of relative species abundance estimates among our collections (*n* = 78) in each habitat category. Our experience has shown that the choice of diversity index can influence the interpretation of differences among communities [40]. To estimate the diversity of each collection and site, we compared two estimators. The Tsallis entropy estimate of diversity (*S*_*q*_) is sensitive to rare species that were expected from incomplete samples and that generally characterise Oak and Wasteland habitat. We also used an extrapolation (i.e., prediction) method that relies on sample completeness and not equal sizes [45] to generate an asymptotic estimator (*D*_*AE*_) of Shannon diversity as implemented in the R package *iNEXT* [45]. Our main hypotheses were concerned with the spatial structuring of species relative abundance and abiotic environmental variation among the sites. By aggregating species relative abundances across the collections by site (Additional file 1: Appendix S1), we essentially removed the temporal signal from data. Though not the focus of this study, we estimated the means and standard deviations in seasonal diversity among the habitats to infer the absence or presence of temporal factors.

### Environmental variation across sites

As the physical locations of wild or managed sites of each habitat category were not grouped by geographic proximity but were intermixed, abiotic environmental variation among sites was not expected to concur with the habitat categories. Generalised linear regression was used to select from 19 climate variables (https://www.worldclim.org/bioclim [46], Additional file 1: Appendix S1), those that were significant in the prediction of the sites from 1000 randomly chosen background points (i.e., pseudo-absences) within the bounding box of the extent of the study. We used topographic variables that comprised raster layers of elevation, aspect, and slope (https://www.europeandataportal.eu). Aspect and slope were calculated from the elevation layer. Land cover variation (spatial polygon; http://centrodedescargas.cnig.es) was included as an indicator of land-use practices, and soil variation [47] as an indicator of historical factors that may influence community structuring. We created a base raster layer of dimensions 720 by 900 cells with resolution of 0.0016° by 0.0016° (the resolution of the elevation layer with the highest resolution) and used this to project the same raster definition to all other layers. The extracted environmental variables were used to construct a site-by-environmental variable matrix used in the subsequent spatial optimisation steps. The highest resolution possible for the WorldClim data was at a spatial resolution of approximately 1 km^2^ (0.0083° by 0.0083°). We calculated pair-wise geographic distances among all the sites and used kernel density estimation to compare the 1 km^2^ grain size with the distribution of distances among the sites (Additional file 1: Appendix S2).

### Spatial optimisation of variables

To demonstrate the difference between variation in diversity and the underlying spatial dependencies among communities, we evaluated spatial autocorrelation among the sites with and without accounting explicitly for the scale of effect. The first spatial autocorrelation approach estimates a global measure of Moran’s *I* [48] from geographic coordinates and species relative abundances, along with one of each of four hypotheses of connectivity used to weight distance relationships between the sites. A Chi-squared transformation of the site-by-species matrix was performed to give weight to rare species. The observed Moran’s *I* [49] was tested against a distribution of values generated by permutation of localities using a Monte Carlo procedure. We conducted a second approach using Moran’s eigenvector maps (MEMs) to capture spatial structures at specific scales among the study sites [35]. Unlike the first approach used to estimate a global measure of autocorrelation, a connectivity graph is combined with an ordination technique to generate spatially optimised explanatory variables, such as for species relative abundance and abiotic environmental variation (Additional file 1: Appendix S1). We chose the MEM eigenfunction approach for spatial optimisation and to estimate Moran’s *I*, because the method allows for a convenient approach to test hypotheses of connectivity using linear connections among sites. A popular approach used in biology to describe geographic connectivity among localities, assumes that various densities of linear connections among points (i.e., study sites) on a planar surface conform to a biological process [50]. The graphs do not explicitly consider information on the influence of landscape connectivity and species movement ecology among the study sites [19], but provide a comparison of more to less restricted connectivity. As the extent of the study included two river valleys, we considered that topographical variation would influence connectivity among the sites. We hypothesised that the hypothesis of connectivity based on Delaunay triangulation represented relatively unrestricted connectivity (i.e., a greater density of edges in the graph) among the sites. The Gabriel graph and the relative neighbour graphs expressed relatively restricted connectivity as hypotheses for the constrained movement of species (Additional file 1: Appendix S1).

The spatially optimised approach uses the Moran’s *I* statistic to evaluate spatial structures and generate MEMs, eigenfunctions that correspond to the *n =* 23 − 1 study sites. Each series of MEMs relates to a different spatial scale. Regression is used to generate an *R*^*2*^ value to detect the maximum amount of variation in the predictor as explained by each MEM (i.e., at each scale). To test the significance of a given MEM, it is necessary to group several of these spatial components within a given ‘smoothing’ window (Additional file 1: Appendix S1). This avoids issues arising from estimation errors expected from too many spatial components [16]. From the 22 MEMs, we smoothed 2 groups of 11 MEMs to what we called the ‘broad’ (MEMs 1–11) and the ‘fine’ (MEMs 12–22) spatial scales respectively. This smoothing scheme had the lowest spatial scale resolution, where more scale categories may have been analysed, but was appropriate to demonstrate spatial dependencies on connectivity. Permutation was used to test if the maximum observed *R*^*2*^ in a group of smoothed MEMs was significantly greater than a randomised distribution. All spatial analyses were conducted in the package *ade4* [51] and with R functions provided by [35] to perform the permutation tests.

### Comparison of diversity and spatial predictors

Linear discriminant analysis (LDA) was chosen to compare the variables in distinguishing the four habitat categories, as this method is suitable for a categorical dependent variable [52]. The approach requires more than one independent variable. In three sets of LDAs we used either the two diversity estimates (*S*_*q*_, *D*_*AE*_), or the three spatially optimised predictors (MEMs). The explanatory power of each of the diversity estimates was expected to be redundant in respect to one another, and in distinguishing the four habitat categories, as they were highly correlated (*r*^*2*^ = 0.944), whereas each of the MEMs were not (*r*^*2*^ < 0.0001). We used MANOVA and a Wilks (λ) post hoc test to see how well the independent variables contributed to distinguishing the habitats. The scale of λ ranges from 0 to 1, where 0 indicates the best discriminatory power. For each set of independent variables, we used either uniform priors (equal probabilities) for the probability of each category, or default priors estimated from the frequency of records in each category. In the third set of LDAs, the data were randomly divided in half to train and test the predictors in a final round of LDAs using the uniform prior. The assumptions of discriminant analysis are multivariate normality, multicollinearity, and variable independence. All data used in the MANOVA and LDA were scaled and centred. Non-normal predictors were pre-processed using Box-Cox transformations as implemented in the R package *caret* [53], to reduce non-normality of the errors and non-linearity in the model.

## Results

### Sample bias and habitat diversity

We were interested in how estimators of biodiversity based on species relative abundance can be used to distinguish habitats. The rarefaction analyses of the collections made on each sampling occasion at a particular site, indicated near asymptotic relationships between the number of samples and the expected number of species (Additional file 1: Appendix S3). A DCA (Additional file 1: Appendix S4) was used to assess the relative homogeneity of the collections among each of the habitats, and supported our categorisation of each habitat. Crop did not form a homogenous habitat category and produced several distinct clusters that depended on the plant assemblies associated with the crop species and their Edge community (Additional file 1: Appendix S5). We used two estimators of diversity to account for rare or dominant species (evenness), and another for incomplete sampling. Differences among the means of the Tsallis entropy (*S*_*q*_) and extrapolated (*D*_*AE*_) estimators of the diversity of each habitat were evident (Additional file 1: Appendix S6, Appendix S7). Differences in diversity among the habitats were sensitive to the choice of diversity estimator when abundances were aggregated at the level of site (Additional file 1: Appendix S8). In general, Crop had the lowest diversity followed by Edge, Oak, and Wasteland with the highest diversity.

### Spatial structuring among sites

**Fig. 2:**
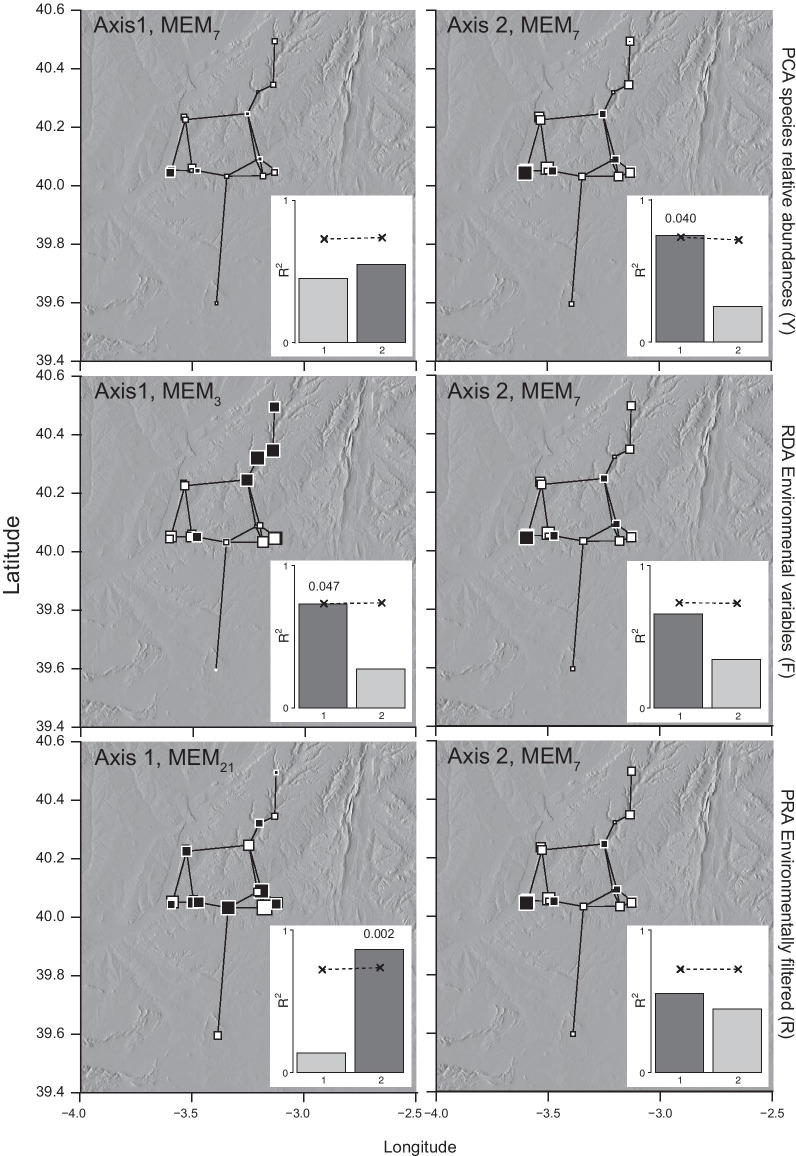
Eigenvalue scores (Moran’s eigenvector maps, MEMs) given by the first and second axes produced by each ordination and connectivity described by the Gabriel graph. The first two axes of each of the ordination analyses of the spatial and environmental variables (matrices, Y, F, R) are shown with the connectivity graph superimposed over the study sites. Insets show permutation tests of sets of smoothed MEMs for the broad (1) and the fine (2) scales. Eigenvalue scores are proportional to the size of the squares (black = negative, white = positive) at each site. The ordination with highest *R*^*2*^ is given in each case. The significance was determined by permutating the maximum observed *R*^*2*^ against a null distribution of the other MEMs in the group having values with no spatial structure. The 95 % quantile of the simulated *R*^*2*^ distribution is indicated with crosses connected by a dotted line with significant *p*-values indicated on inset graphs

Four hypotheses of connectivity (i.e., linear connections among study sites on a planar surface) were used to compare spatial structuring under a range of connectivity scenarios (Table 1). The global estimates of spatial autocorrelation (i.e., with no spatial optimisation) indicated significant (*p* < 0.05) and relatively weak (i.e., close to zero) positive spatial autocorrelation among sites regardless of the hypothesis used to describe connectivity (Additional file 1: Appendix S9). The spatial autocorrelation approach that was sensitive to the scale of effect identified both fine- and broad-scale effects. The permutation of the smoothed groups of MEMs (i.e., either at the fine- or broad- scale) indicated significant structuring (i.e., spatial patterns of distributions of species or environmental variation) among the sites at the broad scale (MEMs 1–11). For instance, the MEMs generated under all connectivity graphs (e.g., Fig. 2; by the Gabriel graph, *p* = 0.031) indicated significant broad-scale structuring of species relative abundances. The Gabriel and Delaunay graphs also generated MEMs that indicated significant (*p* = 0.040 and *p* = 0.024, respectively) broad-scale structuring given the environmental factors. When the signal from the environmental variation was filtered from the species abundance relationships, the Gabriel and Delaunay (Fig. 2, Additional file 1: Appendix S10) connectivity graphs generated significant structuring at fine spatial scales (MEMs 12–22).

**Table 1:** Permutation of the maximum observed *R*^*2*^

		Gabriel graph		Delaunay triangulation		Relative neighbour 1		Relative neighbour 2	
Ordination	Scale	Obs.	p-value	Obs.	p-value	Obs.	p-value	Obs.	p-value
Y Axis 1	Coarse	0.451	0.628	0.420	0.712	0.623	0.209	0.623	0.205
	Fine	0.549	0.373	0.580	0.289	0.377	0.792	0.377	0.796
Y Axis 2	Coarse	0.749	**0.040**	0.780	**0.024**	0.810	**0.013**	0.810	**0.020**
	Fine	0.251	0.961	0.220	0.977	0.190	0.988	0.190	0.981
F Axis 1	Coarse	0.729	**0.047**	0.611	0.205	0.652	0.134	0.652	0.184
	Fine	0.271	0.954	0.389	0.796	0.348	0.867	0.348	0.817
F Axis 2	Coarse	0.661	0.125	0.736	**0.042**	0.585	0.282	0.585	0.292
	Fine	0.339	0.876	0.264	0.959	0.415	0.719	0.415	0.709
R Axis 1	Coarse	0.136	0.999	0.262	0.956	0.406	0.745	0.406	0.747
	Fine	0.864	**0.002**	0.738	**0.045**	0.594	0.256	0.594	0.254
R Axis 2	Coarse	0.556	0.351	0.310	0.881	0.572	0.295	0.572	0.329
	Fine	0.444	0.650	0.690	0.120	0.428	0.706	0.428	0.672

Significant tests are indicated with bold text

The spatial variables are for the first and second axes of a PCA of site-by-species (**Y**, 0.167 and 0.162 of total variance), an RDA of the environmental variables (**F**, 0.202 and 0.198 of total variance), and a partial residual analysis (**R**, 0.239 and 0.195 of total variance). Significant tests indicated with bold text. The standardised observations are calculated by subtracting the mean of the randomised values and dividing by their standard deviation

Significant and positive spatial autocorrelation was detected at the broad-scale regardless of the hypothesis of connectivity (Table 2). For example, evidence of significant spatial structuring and positive spatial autocorrelation at the broad scale corresponded to MEM_7_ given the Gabriel graph (Moran’s *I* = 0.336, *p* = 0.023). However, significant fine-scale structuring and negative spatial autocorrelation was dependent on the connectivity graph (Additional file 1: Appendix S11, Appendix S12). Significant negative spatial autocorrelation at the fine-scale was evident when the Gabriel and Delaunay graphs were used, which hypothetically described landscape connectivity that conformed to the river valleys.

**Table 2:** Permutation tests of the observed Moran *I* of MEMs with the maximum *R*^*2*^ of a given ordination (Y, R, F) axis (e.g., see Fig. 2). Significant tests are indicated with bold text

SWM	Test	Obs.	Exp.	Var.	H_0_	*p*-value
Gabriel graph	MEM_3_	0.670	− 0.044	0.170	Two-sided	**0.001**
	MEM_7_	0.336	− 0.044	0.165	Two-sided	**0.023**
	MEM_21_	− 0.840	− 0.040	0.173	Two-sided	**0.001**
Delaunay triangulation	MEM_4_	0.428	− 0.045	0.115	Two-sided	**0.001**
	MEM_5_	0.297	− 0.044	0.117	Two-sided	**0.005**
	MEM_9_	− 0.017	− 0.051	0.115	Two-sided	0.784
	MEM_13_	− 0.236	− 0.047	0.113	Two-sided	0.090
	MEM_18_	− 0.394	− 0.048	0.115	Two-sided	**0.007**
Relative neighbour 1	MEM_4_	0.630	− 0.026	0.155	Two-sided	**0.001**
	MEM_6_	0.381	− 0.040	0.139	Two-sided	**0.004**
	MEM_8_	0.272	− 0.032	0.147	Two-sided	**0.036**
	MEM_10_	− 0.107	− 0.042	0.153	Two-sided	0.679
Relative neighbour 2	MEM_4_	0.590	− 0.038	0.157	Two-sided	**0.001**
	MEM_6_	0.433	− 0.042	0.153	Two-sided	**0.001**
	MEM_8_	0.290	− 0.035	0.155	Two-sided	**0.032**
	MEM_10_	0.138	− 0.039	0.151	Two-sided	0.242

Significant tests are indicated with bold text

### Spatially optimised versus diversity variables

**Fig. 3:**
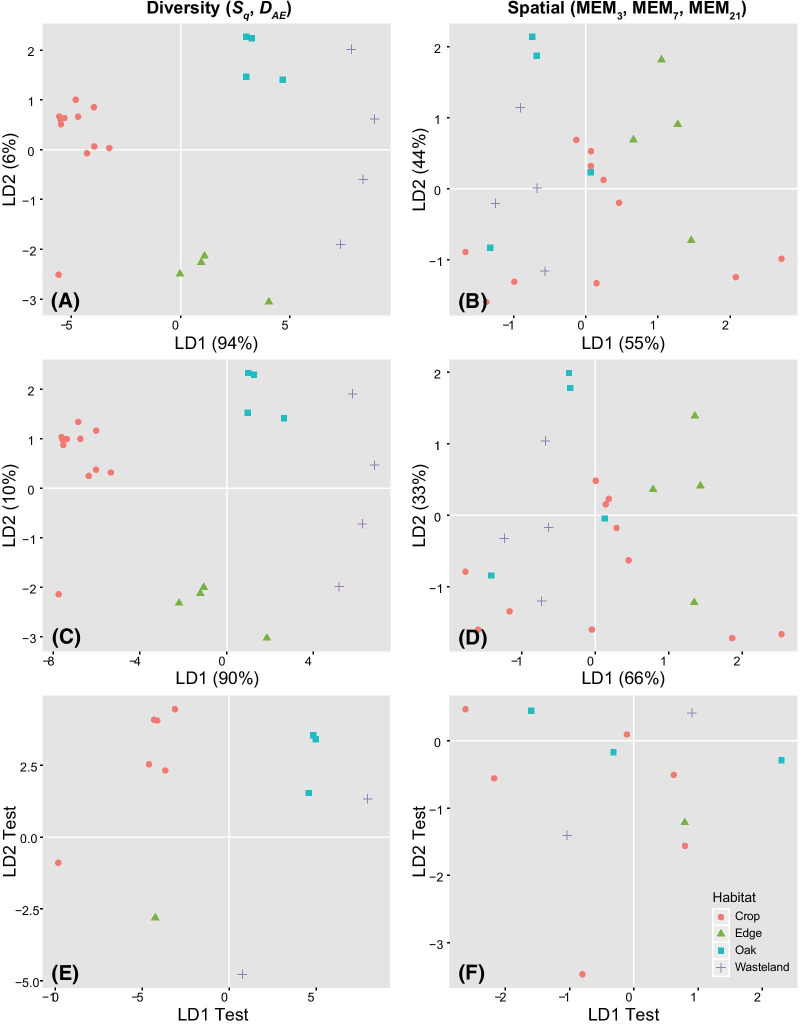
Linear discriminant analysis (LDA) of diversity (*S*_*q*_ and *D*_*AE*_; **A**, **C**, **E**) and spatial predictors (MEM_3_, MEM_7_, MEM_21_; **B**, **D**, **F**) of the habitat categories. The top two plots show LDA predictions using uniform priors (equal probabilities) for the probability of each category, the middle plots were generated using priors estimated from the frequency of records in each category, and the plots on the bottom were from a random test set of predictors

We tested the hypothesis that species diversity differences among habitats (Additional file 1: Appendix S4) are subject to spatial dependencies. The diversity estimates (*S*_*q*_ and *D*_*AE*_ at each site) were compared to the spatially optimised predictors (Gabriel SWM; MEM_3_, MEM_7_, MEM_21_) in their ability to recover distinctions among the four habitats (Fig. 3). A Wilks post hoc test of the MANOVA (Table 3) was significant and rejected the null hypothesis of equality of habitat means [Wilks λ = 0.011, *F*(6, 36) = 52.34, *p* < 0.0001] when the diversity estimates were used to distinguish the four habitats. By comparison, the spatially-optimised variables produced non-significant [Wilks λ = 0.011, *F*(9, 41.52) = 1.74, *p* = 0.111] differences among the means. The LDAs grouped the diversity estimates of each site by habitat, but these distinctions were not clear when the spatially optimised predictors were used. The presence of underlying spatial structuring, and collinearity among the diversity estimates for each site of each habitat, implies that high or low diversity does not represent a level that corresponds to specific biological processes. The spatial variables informed on underlying processes that connected the study sites, while species diversity only informed on distinctions among the four habitat categories.

**Table 3:** Multivariate analysis of variances (MANOVA) showing the variance relationships among the spatial and diversity variables used to predict the habitat categories

	D.f.	Sum Sq.	Mean Sq.	*F*-value	*P*(> *F*)
*S*_*q*_ ~ Habitats	3	27.75	9.25	153.69	> 0.0001
Residuals	19	1.14	0.06		
*D*_*AE*_ ~ Habitats	3	26.38	8.79	76.64	> 0.0001
Residuals	19	2.18	0.11		
MEM_3_ ~ Habitats	3	5.53	1.84	2.16	0.127
Residuals	19	16.23	0.85		
MEM_7_ ~ Habitats	3	5.92	1.97	2.03	0.144
Residuals	19	18.47	0.97		
MEM_21_ ~ Habitats	3	3.83	1.28	1.13	0.363
Residuals	19	21.53	1.13		

## Discussion

The explicit consideration of connectivity and spatial structuring among study sites is critical when predicting biological processes in heterogeneous landscapes [5]. The use of spatially explicit variables is not only crucial for correcting statistical analyses for spatial autocorrelation [15], but might provide a useful surrogate for biological processes that are difficult or impossible to measure [9]. The results showed both broad- and fine-scales of effect on the structuring of species relative abundances among the sites. Conversely, the species diversity indices did not inform on the influence of the abiotic environmental variation (the broad-scale) and the configuration of plant assemblages (the fine-scale). Importantly, these scales of effect were dependent on the hypothesis that weighted connectivity among the sites. These findings suggest that spatially optimised variables that incorporate a priori information on connectivity, inform on underlying processes that connect communities [13, 30]. Information on unmeasured broad- and fine-scale processes that may influence plant assembly and local community structure, will therefore elucidate on the connection between demographic stochasticity among communities and the potential for dispersal among them.

### Connectivity and spatial structuring among sites

We hypothesised that the scale of effect is dependent on the connectivity among communities, i.e., densities and conformations of linear connections among sites. Our findings showed that significant spatial structuring was concomitant with positive spatial autocorrelation (i.e., similar values cluster over the study area) of species relative abundances at the broad scale for all four hypotheses of connectivity (Tables 1 and 2). However, significant negative spatial autocorrelation (i.e., large disparities between the values of neighbouring sites) at fine scales (Table 2) was associated with the less restrictive expressions of connectivity (i.e., a relatively high density of connections among sites). Broad-scale spatial effects have conventionally been interpreted as resulting from environmental drivers, while structuring at fine scales and negative spatial autocorrelation has been interpreted as a consequence of proximity among sites with dissimilar attributes [35]. Dispersal pathways and species interactions, are predicted to respond differently to the influence of spatial autocorrelation [54]. Although we cannot objectively assess the performance of our hypotheses for connectivity without data such as species movement ecology, the findings demonstrate that it is critical to account accurately for connectivity among communities, in order to identify the scale of effect that is most meaningful to the processes being investigated.

The application of canonical approaches such as using a Gabriel graph to describe connectivity, has been attractive to biologists as seen in its many applications [55]. However, it is questionable whether approaches that are based on linear lattice relationships, as we have used here, accurately represent descriptions of species movements in heterogeneous landscapes. For example, an alternative approach weighted air passenger movement rates by infection prevalence to generate malaria risk maps among locations in a global air travel network [56]. Another approach corrected distances by accounting for the total overland distance between sites imposed by geographic topology [57]. Additionally, in our study we did not consider the role intervening communities may have had on connectivity bias among the sites. For example, Thompson and colleagues [27] simulated habitat loss by removing local communities that served as connections among other communities. The study showed that the removal of communities disrupted dispersal among them and affected ecosystem diversity, function, and stability. It may be the case that hypotheses of connectivity are sensitive to variation in dispersal at different periods throughout the year. However, determining such cycles is beyond the scope of this study.

The importance of spatial structuring in modifying community dynamics cannot be understated. Connectivity and spatial structuring are expected to influence coevolutionary processes between plants and their associates, and adaptation to new niches, especially when preferences are not strongly constrained by species-specificity (e.g., [58–60]). Community composition is ultimately linked to the competing influences of environmental filtering (i.e., the optimisation of living conditions) and mechanisms (e.g., competition, mutualism) permitting coexistence [61, 62]. The spatial arrangement and connectivity of communities’ influences species movements among them and interactions within, and hence, their functional diversity.

### Spatially optimised versus diversity variables

Our second hypothesis proposed that diversity estimators used to characterise functional relationships are subject to spatial structuring and abiotic environmental variation among the sites. Both diversity estimates were redundant, but were consistently good at recovering significant clusters of sites of each habitat category regardless of the prior used for the analysis (Table 3; Fig. 3A, C). Even with the relatively few records of sites that were available for some of the habitats, the supervised LDA prediction using the diversity estimates also recovered each category (Fig. 3E). By contrast, the LDA plots generated from the spatially optimised variables showed no redundancy, with notable distinctions between the Crop and Wasteland sites only, and diffuse and overlapping clusters among them and the sites of Oak and Edge (Fig. 3B, D). The predictive LDA with the spatial predictors showed the same pattern (Fig. 3F) of non-significant variance relationships among the habitat categories (Table 3). Wasteland sites were over-dispersed in the ordination space, with some sites more similar to Oak and some more to Edge sites. It would therefore be prudent to introduce either of the species diversity estimates, as a measure of distinctions among communities, as well as spatial predictors that relate to processes that connect them, to test their respective contributions to model variance. Furthermore, our cursory analysis of temporal effects on species diversity of communities (Additional file 1: Appendix S8) suggests that seasonal variation may be associated with the density and the reproduction cycles [63] of plant species [58]. Altogether, the spatial scale-optimisation approach that we used here will be extendible to understanding the scale at which species traits [64] or resource use [65] contribute most strongly to response variables or residuals in dynamic models.

## Conclusions

Key challenges to modelling drivers of biodiversity include linking biological processes with functional features of species diversity [66]. Linking the spatial distributions of resources of multiple species with the particular spatial (or temporal) resolutions at which these associations are most meaningful [reviewed in 67] is crucial to predictive modelling [68]. We show that explanatory factors hypothesised to drive biological processes can be assigned to spatially discrete scales. The spatial structuring among plant communities relied on the type of connectivity among communities, which should ideally be quantified using information of species movement ecology. The findings show that the diversity estimators used to characterise plant communities, may not provide information about biological processes if the study does not connect processes of interest with the scale(s) of effect. Spatially optimised predictors have the potential to relate multiple unmeasurable variables to biological processes, and at the very least, provide a means of testing whether spatial factors contribute to statistical variance or error. Trait responses to spatial dependencies of many co-occurring species may now be compared in downstream analyses. Future attention to better characterising spatial dependencies among traits of communities will also improve models of biological processes.

## Supplementary Information

**Additional file 1.** Additional details of study area and spatial optimisation method.

## Data Availability

The datasets generated and/or analysed during the current study are available from the Dryad Digital Repository (available at, 10.5061/dryad.s7h44j13b).

## Abbreviations

SWM: Spatial weighting matrix
MEM: Moran’s eigenvector map
DCA: Detrended correspondence analysis
D_AE_: Asymptotic estimator of Shannon diversity
UTM: Universal Transverse Mercator
PCA: Principal component analysis
RDA: Redundancy analysis
PRA: Partial residual analysis
LDA: Linear discriminant analysis
MANOVA: Multivariate analysis of variance
