# Supplementary material for: Structuring of plant communities across agricultural landscape mosaics: the importance of connectivity and the scale of effect
Source: BMC Ecol Evol. 2021 Sep 9;21:173. doi: 10.1186/s12862-021-01903-9 (PMC8427894; doi:10.1186/s12862-021-01903-9)
Supplement: Supplementary file 1 — Additional file 1. Additional details of study area and spatial optimisation method. [file 12862_2021_1903_MOESM1_ESM.docx]

Additional file 1

**Appendix S1.**

**Study sites used for sampling**

The study area is situated in the Mediterranean Basin, one of 25 global biodiversity hotspots (Myers et al., 2000). The regional climate is classified as Mediterranean, with strong seasonal and semiarid characteristics of warm-dry summers and mild-humid winters (Roquero et al., 2015). The area experiences summer soil-water deficits, although arable areas are extensively irrigated (Lorenzo-Lacruz et al., 2010). The Tagus River valley is in a mountainous area. The average elevation of study sites (*n* = 23) was 595 m (range: 490 m to 881 m). The mean annual temperature within the extent of the study was 13.4 °C (10.5 °C to 15.3 °C) with a mean annual precipitation of 440 mm (range: 378 mm to 530 mm). The fluvial soils of the Tagus River valley developed on limestone, marl, gypsum, and clays (Roquero et al., 2015). Forested sites are characterised by sandy neutral to slightly acidic soils and are not suited to agriculture because they tend to be situated in rocky terrain (Plieninger et al., 2003).

The vegetation cover types that discriminated our *a priori* habitat categories, represent historical and physical characteristics of agro-ecosystems in central Spain. Each of these cover types were affected by different intensities of anthropic disturbance. Evergreen oak forests (Oak) are considered natural ecosystems that have been variably disturbed by practices such as fuel production (prior to 1950s), for recreational reserves, or light grazing. These areas are dominated by *Quercus* species with understory vegetation comprised of tall sclerophyllous shrubs and grasses (Plieninger et al., 2003). Patchy areas of successional scrubland of no particular use (Wasteland) are interspersed among crop areas. The abandonment of traditional farming and the adoption of modern practices has increased the patchiness of successional scrubland (Wasteland) and woodland communities (Romero-Calcerrada & Perry 2004). Crops (Crop) are largely annual monocultures interspersed by weed species, which are rotated or left fallow between seasons. Crops are separated by narrow borders of relatively permanent plant assemblages (Edge) generally left intact between seasonal cropping. Edge sites occasionally experience low-level disturbance either by burning or partial ploughing. The rationale for our sampling strategy was based on the variation of the configuration and area of the cover types associated with each habitat. This variation required different sampling efforts between habitats to be confident of a reasonable estimation of species richness. The number of repeat (‘replicate’) sites and re-sampling at sites of each habitat at different time points was chosen as a consequence of meeting a balance between the ability to capture within-habitat (seasonal) variability and addressing the practicalities related to crop rotation, access to sites, cost, and time.

Other cover types present within the extent of the study comprise road margins, domestic gardens, and industrial sites that are intermittently dispersed near towns or villages. Olives and vines are typically cultivated in drier habitat where oaks once grew, and were not included in this study. Olive groves and vineyards are subject to regular ‘cleaning’ of successional ‘invasions’. In one instance, an Edge site (L2P2) was cleared of vegetation and not sampled in that season. Access permission to some sites (M2, M6, M10) was not possible during some seasons, and an alternative, compatible site was chosen and sampled.

**Habitat diversity and data aggregation**

As we used a repeat sampling strategy (i.e. multiple collections at the same site) and sampled multiple sites of the same habitat, we evaluated the effect of data aggregation known to influence parameter estimates. The distinctions between Edge and Oak were most pronounced when the *D_AE_* estimator was used and when the abundance data were aggregated (Additional file 1: Appendix S8). Conversely, when the *S_q_* estimator was used without data aggregation, the relationship between Edge and Oak was reversed.

**Environmental predictors**

For the purposes of illustrating environmental (i.e. spatial) dependencies among the sampling sites, we used freely available online data, and acknowledge that other variables may be more appropriate for the fine-scale analyses (e.g. land cover, site-specific soil pH, nutrient profiles, etc). We used topographic variables that comprised raster layers of elevation, aspect, and slope (<https://www.europeandataportal.eu>). Aspect and slope were calculated from the elevation layer. The spatial polygon object of land cover variation is available from (<http://centrodedescargas.cnig.es>). A generalised linear model was used to select from the 19 WorldClim climate variables that best explained the distribution of the study sites (https://www.worldclim.org/bioclim):

BIO1 = Annual Mean Temperature
BIO2 = Mean Diurnal Range (Mean of monthly (max temp - min temp))
BIO3 = Isothermality (BIO2/BIO7) (* 100)
BIO4 = Temperature Seasonality (standard deviation *100)
BIO5 = Max Temperature of Warmest Month
BIO6 = Min Temperature of Coldest Month
BIO7 = Temperature Annual Range (BIO5-BIO6)
BIO8 = Mean Temperature of Wettest Quarter
BIO9 = Mean Temperature of Driest Quarter
BIO10 = Mean Temperature of Warmest Quarter
BIO11 = Mean Temperature of Coldest Quarter
BIO12 = Annual Precipitation
BIO13 = Precipitation of Wettest Month
BIO14 = Precipitation of Driest Month
BIO15 = Precipitation Seasonality (Coefficient of Variation)
BIO16 = Precipitation of Wettest Quarter
BIO17 = Precipitation of Driest Quarter
BIO18 = Precipitation of Warmest Quarter
BIO19 = Precipitation of Coldest Quarter.

Six of the 19 WorldClim climate variables had significant effects: Annual Mean Temperature, Mean Temperature Diurnal Range, Temperature Seasonality, Minimum Temperature of Coldest Month, Precipitation Seasonality, Precipitation of Coldest Quarter. These were combined with aspect, elevation, slope, soil type (The European Soil Database distribution version 2.0, 2004; The European soil database, 2006), and land-cover to assess environmental differences among the sites in the spatial-optimisation step.

**Spatial weighting matrices**

Geographic-connectivity graphs describe pairs of points connected by lines to provide a representation of adjacent localities. Two canonical and two custom neighbour-connectivity graphs were used to generate spatial weighting matrices (SWM) that assign weights proportional to a function of geographic distance between neighbouring sites, and express hypotheses of plausible transmission pathways among the sites. The connectivity graphs comprised neighbour matrices generated using Delaunay triangulation (the maximum density of linear connections) and a Gabriel graph. Two custom neighbour graphs were also generated manually by editing a relative neighbour graph (minimum density of connections) with linear connections that conformed to the major river valleys present in the study area. The custom graphs were designed to concur with site connectivity being driven by the river valleys, and the degree of connectivity the most isolated site would have with the others. These pathways were assumed to arise as a consequence of wind channelling, elevational temperature differential (between Oak and the other habitats), predator and herbivore (of e.g., vectors, pollinators, parasites, and hosts) diversity outside the crop habitats (e.g. Álvarez et al., 2019). The connectivity described by the custom graphs was adjusted to produce a symmetric SWM required for matrix operations. Each neighbour graph was weighted by a function of geographic (Universal Transverse Mercator, UTM) distances between sites (1 minus each distance relationship divided by the maximum distance observed), which assumes that connectivity decreases with distance.

**Spatial analysis of smoothed MEMs**

We also grouped 3 groups of 7 Moran’s eigenvector maps (MEMs) to generate an intermediate scale. No intermediate scale significances were produced and we chose to use the 2 x 11 grouping arrangement. When a significant MEM has been identified, it is used as a spatially-optimised explanatory variable. Due to the right-tail skew caused by the relatively high proportion of rare species expected from incomplete samples (Chao et al., 2014), a Chi-squared transformation of the site-by-species matrix is recommended to give more weight to rare species in the data. The MEM eigenfunctions correspond to the (23 - 1) study sites and are divided into subsets of positive and negative eigenvalues (positive and negative spatial autocorrelation respectively). The ordination scores of the first and second axes of a principal component analysis (PCA) of the site-by-species matrix (Y), a redundancy analysis (RDA) of the site-by-species relationships given environmental variation (F), and a partial residual analysis (PRA) used to model the effects (a filter of the orthogonal instrumental variable) of the environment on the residuals of species relative abundances (R), were then each regressed on the MEMs of the four connectivity hypotheses.

The Figures (Figs. 2, S7-S9) that show the SWM relationships with the study sites, the eigenvalue scores given by the first and second axes produced each of the Y, F, and R matrices and Gabriel graph are interpreted as follows. The ordination scores are represented by either black (positive) or white (negative) squares proportional to the value of the score. The insets show scalograms of permutation tests of the maximum *R^2^* produced from the smoothed sets of MEMs, which indicate the spatial scale at which the structuring was significant or not. Spatially random black and white scores are indicative of negative spatial autocorrelation (e.g. Fig. 2, Axis 1, MEM21, PRA), while either spatially uniform black or white scores are interpreted as areas of positive spatial autocorrelation (e.g. Fig. 2, Axis 1, MEM3 of the RDA).

**References**

Álvarez, H. A., Morente, M., Oi, F. S., Rodríguez, E., Campos, M., and Ruano, F. (2019). Semi-natural habitat complexity affects abundance and movement of natural enemies in organic olive orchards. Agriculture, Ecosystems & Environment 285, 106618.

Chao, A., Gotelli, N. J., Hsieh, T. C., Sander, E. L., Ma, K. H., Colwell, R. K., and Ellison, A. M. (2014). Rarefaction and extrapolation with Hill numbers: a framework for sampling and estimation in species diversity studies. Ecological Monographs 84, 45-67.

Lorenzo-Lacruz, J., Vicente-Serrano, S. M., López-Moreno, J. I., Beguería, S., García-Ruiz, J. M., Cuadrat, J. M. (2010). The impact of droughts and water management on various hydrological systems in the headwaters of the Tagus River (central Spain). Journal of Hydrology 386, 13-26.

Myers, N., Mittermeier, R. A., Mittermeier, C. G., Da Fonseca, G. A., Kent, J. (2000). Biodiversity hotspots for conservation priorities. Nature 403, 853-858.

Plieninger, T., Pulido, F. J., and Konold, W. (2003). Effects of land-use history on size structure of holm oak stands in Spanish dehesas: implications for conservation and restoration. Environmental Conservation 30, 61-70.

Romero-Calcerrada, R., and Perry, G. L. (2004). The role of land abandonment in landscape dynamics in the SPA ‘Encinares del rı́o Alberche y Cofio, Central Spain, 1984–1999. Landscape Urban Planning 66, 217-232.

Roquero, E., Silva, P. G., Goy, J. L., Zazo, C., & Massana, J. (2015). Soil evolution indices in fluvial terrace chronosequences from central Spain (Tagus and Duero fluvial basins). Quaternary International, 376, 101-113.

The European soil database (2006) GEO: connexion, 5 (7), pp. 32-33.

The European Soil Database distribution version 2.0, European Commission and the European Soil Bureau Network, CD-ROM, EUR 19945 EN, 2004.


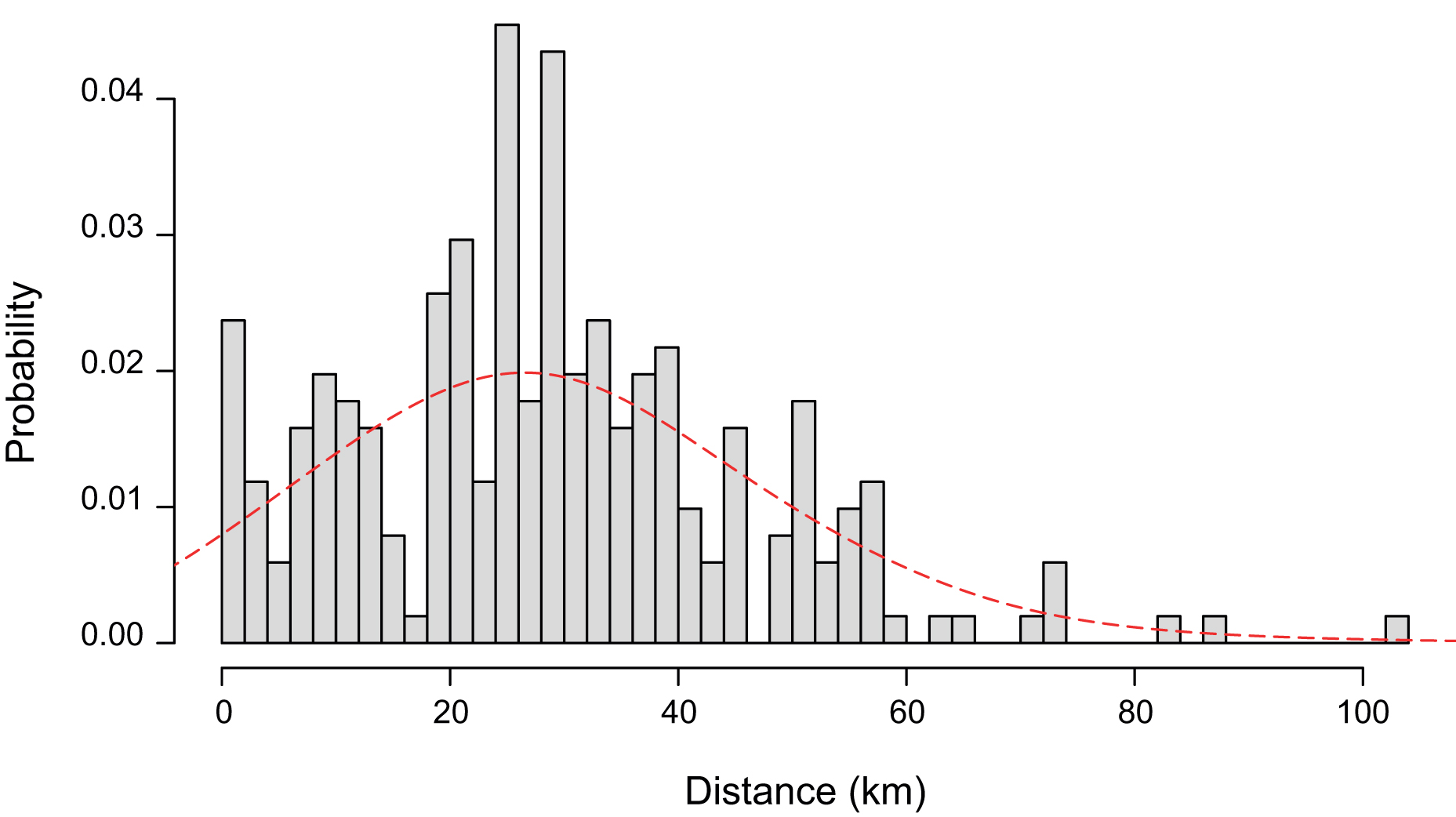


**Appendix S2.** A frequency (probability) distribution of pair-wise distances among the sampling sites (*n* = 23) used in the study. The mean distance between sites was 28.8 km. The red dashed line was fitted to the distribution using kernel density estimation with a Gaussian smoothing function. The analysis of pair-wise distances indicated that the 1 km^2^ resolution of the WorldClim data was relatively fine compared to the distances between a large majority of the sites. This indicated that if there was environmental variation between adjacent 1 km^2^, or between locations farther apart, then these differences would be reflected at the resolution of the study for most of the sites. There was a small proportion of sites that were less than 2 km apart, and presumably have similar climatic and abiotic characteristics.

**Appendix S3.** Rarefaction curves of species richness for individual sites (*n* = 78) of the study. Vertical lines indicate the respective sample sizes. Vertical lines indicate the respective sample sizes. The first two characters of the site codes indicate the habitat affiliation: (Cr) Crops either of (B) *Brassica* *oleracea*, (Z) *Zea mays*, (C) *Cucumis melo*, (H) *Hordeum* *vulgare*; (Ed) Edge; (Wl) Wasteland; and (Oa) evergreen Oak forest. The numeric characters are the ‘replicate’ site and order of sample date. Coloured rarefaction curves are random to aid visualization.


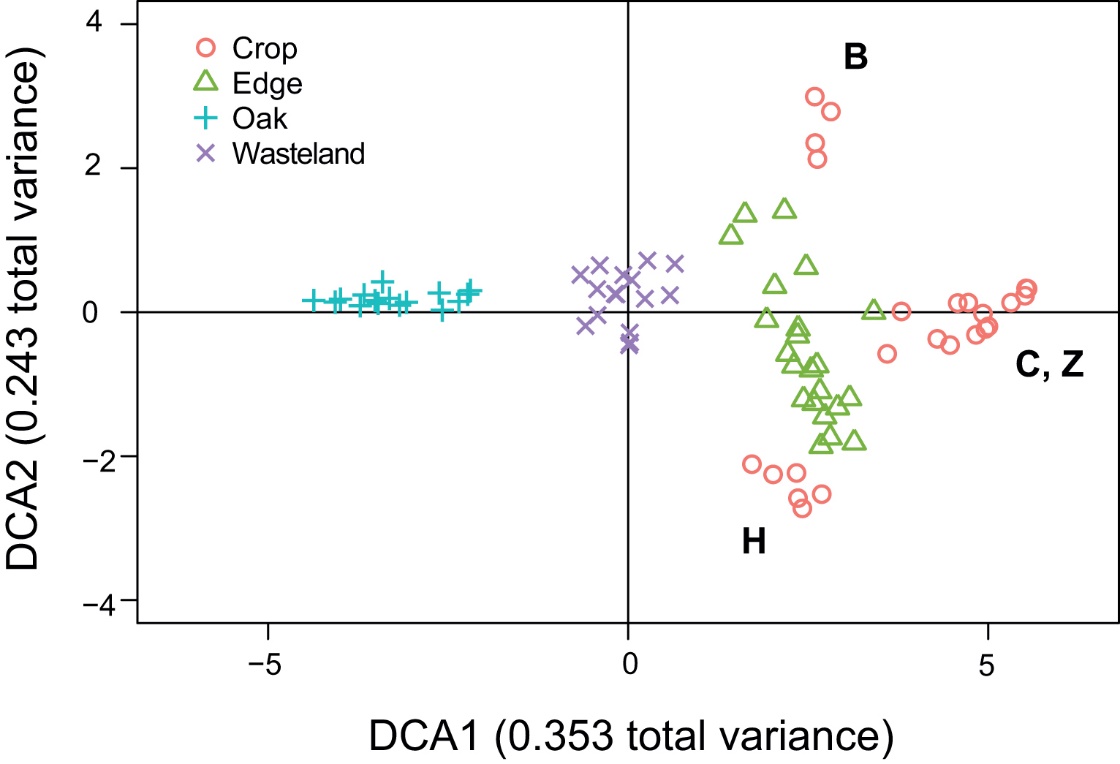


**Appendix S4.** Detrended correspondence analysis (DCA) of site by species abundance matrix collected from the four habitat categories. Each point in the graph represents all the samples collected on a single occasion at a particular site of the study. Analysis of species abundances was at the collection (*n* = 78) level. The bold letters highlight collection from Crops: (B) *Brassica oleracea*, (Z) *Zea mays*, (C) *Cucumis melo*, (H) *Hordeum* *vulgare*.


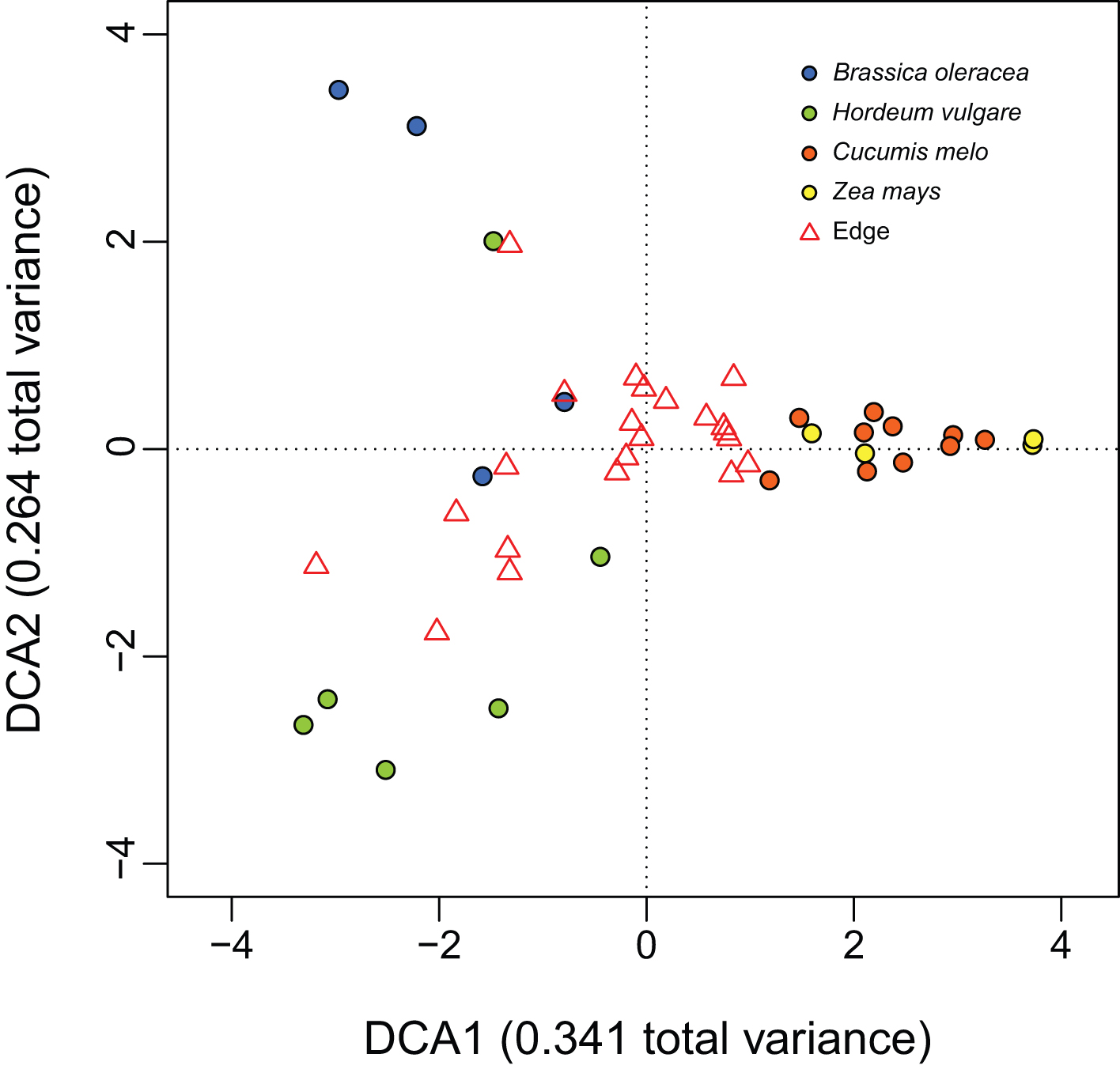


**Appendix S5.** Detrended correspondence analysis of Edge and Crop collections. The dominant crop species was removed from the Crop dataset to assess clustering without their strong influence on the ordination scores.

**Appendix S6.** Tsallis entropy (*S_q_*) and Shannon asymptotic (*D****_AE_***) estimates of diversity. A Levene’s test rejected the null hypothesis of equal homogeneity of variances among the collections (*F*_(3,74)_ = 3.189, *p* = 0.029) and. Kruskal-Wallis rank sum tests indicated significant differences in diversity between habitats at the collection level (*S_q_* *χ*^2^_(3)_ = 54.832, p < 0.001; *D_AE_* *χ*^2^_(3)_ = 43.558, p < 0.001) or sites (*S_q_* *χ*^2^_(3)_ = 18.598, *p* < 0.001; *D_AE_* *χ*^2^_(3)_ = 18.978, p < 0.001). However, when the abundance values were aggregated by site, the Levene’s test accepted the null hypothesis of homoscedasticity. A one-way ANOVA indicated significant differences (*F*_(3,19)_ = 102.00, *p* < 0.0001) in diversity among the habitats. A Tukey Honest Significant Difference test indicated all pair-wise habitat contrasts were significantly different (*S_q_* *p*-adjusted < 0.005) except between Edge and Oak (*S_q_* *p*-adjusted = 0.999).

| **Aggregation** | **Habitat** | ***n*** | ***S_q_ μ* (*σ*)** | *D****_AE_ μ* (*σ*)** |
| --- | --- | --- | --- | --- |
|  |  |  |  |  |
| None | Crop | 24 | 1.481 (0.761) | 5.578 (2.932) |
|  | Edge | 22 | 3.085 (0.533) | 13.648 (3.983) |
|  | Oak | 16 | 4.236 (1.225) | 16.502 (6.940) |
|  | Wasteland | 16 | 4.545 (1.188) | 18.601 (9.166) |
|  |  |  |  |  |
| Site | Crop | 11 | 1.946 (0.889) | 7.891 (4.646) |
|  | Edge | 4 | 6.551 (0.811) | 33.531 (4.767) |
|  | Oak | 4 | 6.470 (0.412) | 24.861 (3.003) |
|  | Wasteland | 4 | 8.653 (0.316) | 40.404 (4.376) |


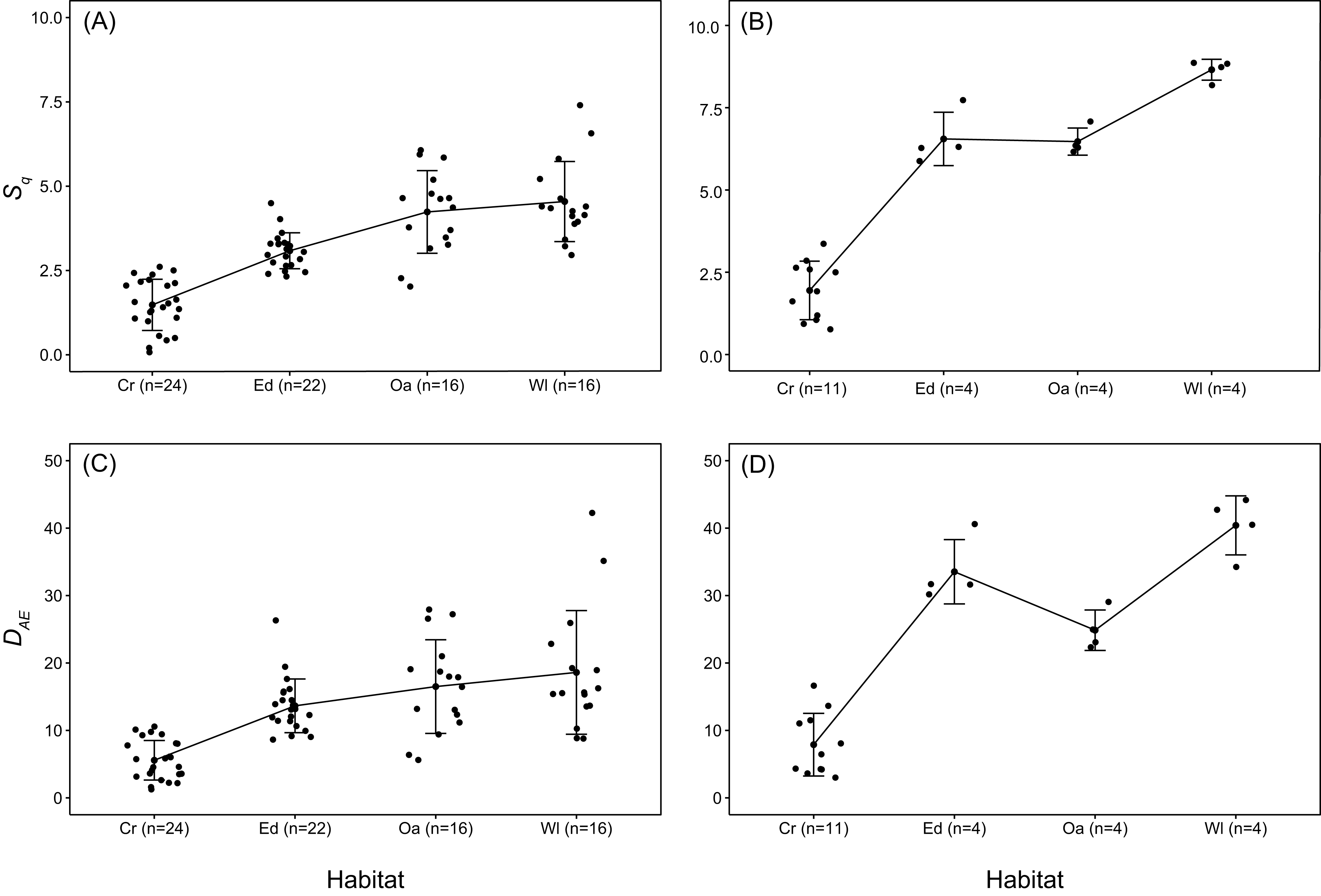


**Appendix S7.** Tsallis diversity (*S_q_*) and Shannon diversity asymptotic estimators (*D_AE_*) means and standard deviations of each habitat. The plots show estimates as calculated from samples of each collection (*n* = 78; A, C) and aggregated by site (*n* = 23; B, D). Abbreviations; Crop (Cr), Edge (Ed), Oak (Oa), and Wasteland (Wl). When the *D_AE_* estimator of ‘true diversity’ was calculated from the aggregated abundance values, there was a larger distinction (*p*-adjusted = 0.052) between Edge and Oak.


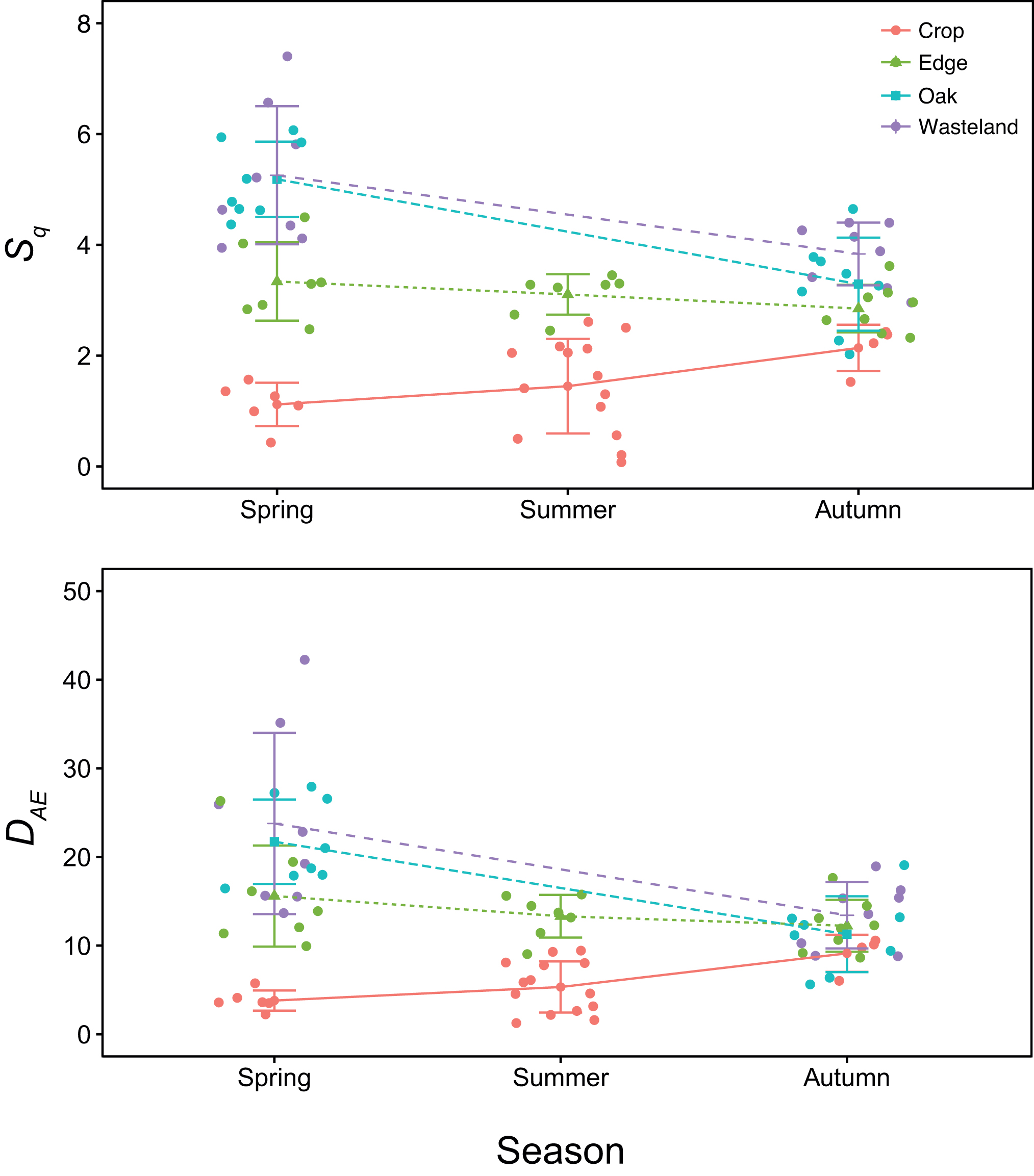


**Appendix S8.** Seasonal variation of the asymptotic (*D_AE_*) and Tsallis entropy (*S_q_*) estimates of diversity among collections (*n* = 78) from each habitat. Jitters indicate mean and standard deviation. There were significant (Kruskal-Wallis rank sum test: *S_q_* *χ*^2^_(2)_ = 18.61, *p* < 0.0001; *D_AE_* *χ*^2^_(2)_ = 17.234, *p* < 0.0002) differences in diversity among seasons.

**Appendix S9** Multiple variate analyses of Moran’s *I* for spatial autocorrelation using each neighbour-connectivity graph. Significant spatial autocorrelation indicated with bold text.

| **SWM** | **Obs. Moran’s *I*** | **Std. obs.** | **Exp.** | **Var.** | ***p*** |
| --- | --- | --- | --- | --- | --- |
| Delaunay | 0.028 | 1.956 | -0.047 | 0.001 | **0.047** |
| Gabriel | 0.050 | 2.044 | -0.043 | 0.002 | **0.049** |
| Relative 1 | 0.053 | 2.145 | -0.047 | 0.002 | **0.044** |
| Relative 2 | 0.053 | 2.141 | -0.046 | 0.002 | **0.040** |


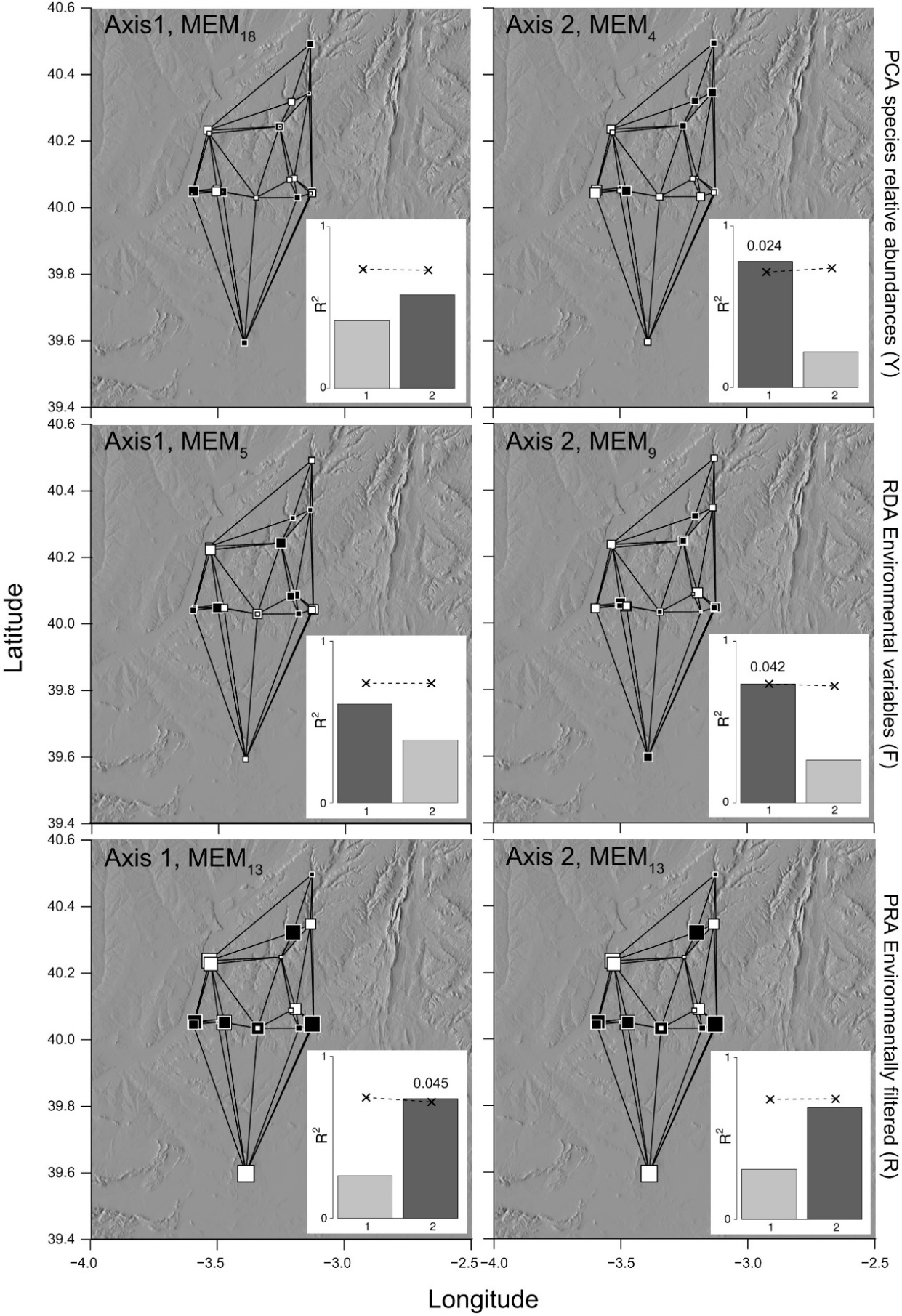


**Appendix S10.** The Delaunay triangulation used to construct spatial weighting matrices for spatial analyses. Insets show permutation tests of sets of smoothed scalograms for the broad (1) and the fine (2) scales. The 95% quantile of the simulated *R^2^* distribution is indicated with crosses connected by a dotted line and significant *p*-values indicated on graph. Eigenvalue scores (black squares) of the ordination with highest *R^2^* given in each case.


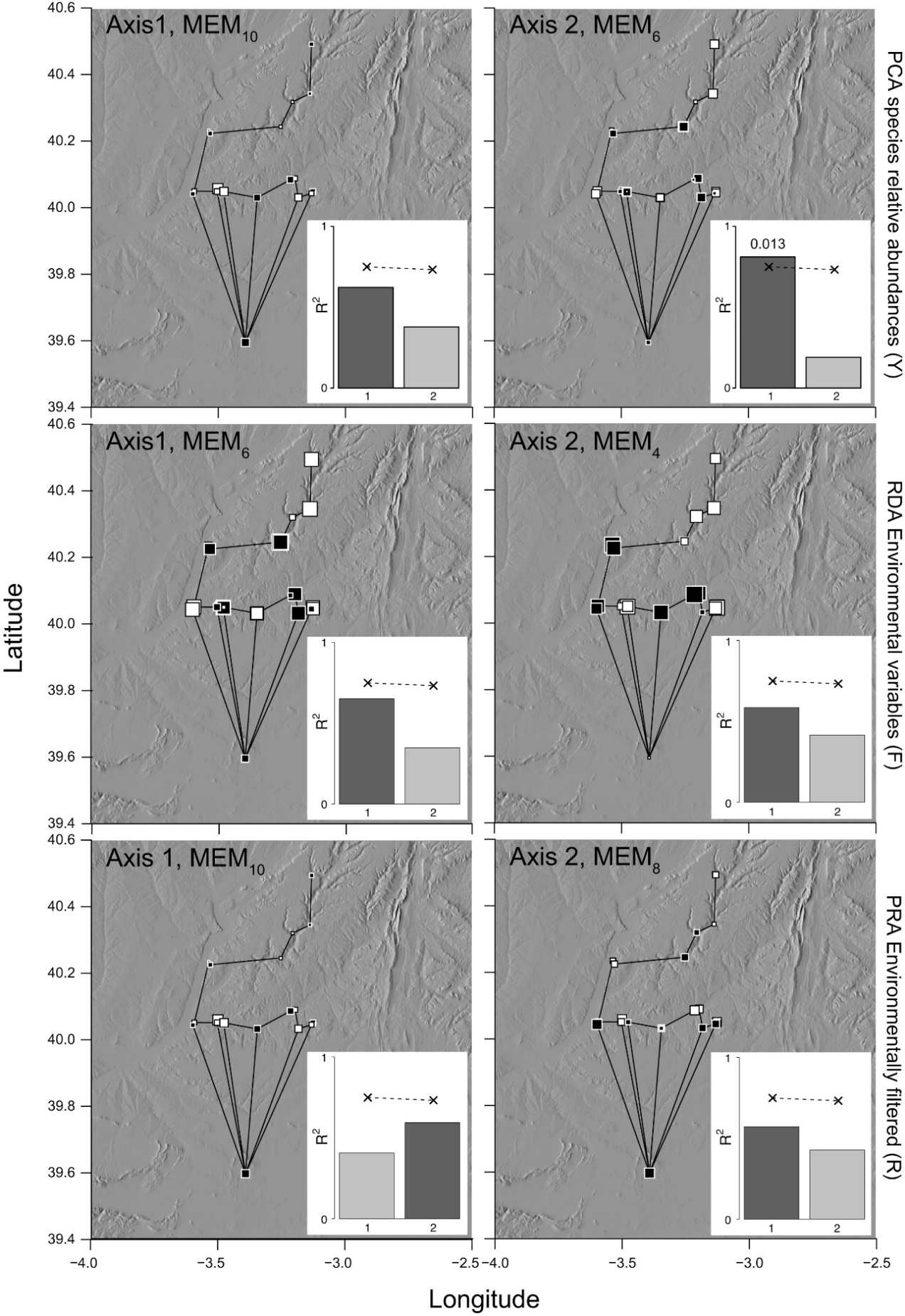


**Fig. S11.** The relative neighbour 1 graph used to construct spatial weighting matrices for spatial analyses. Insets show permutation tests of sets of smoothed scalograms for the broad (1) and the fine (2) scales. The 95% quantile of the simulated *R^2^* distribution is indicated with crosses connected by a dotted line and significant *p*-values indicated on graph. Eigenvalue scores (black squares) of the ordination with highest *R^2^* given in each case.


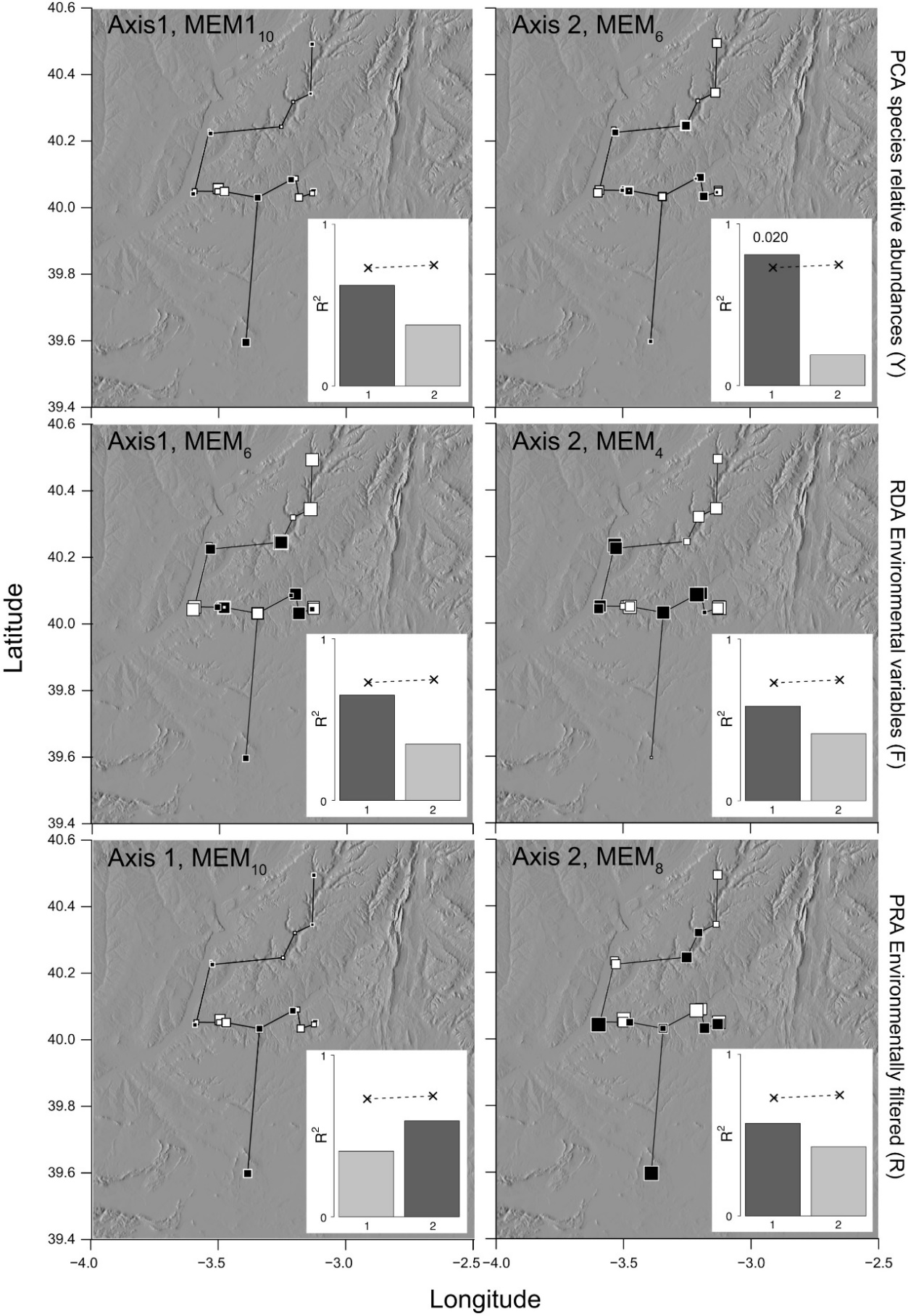


**Fig. S12.** The relative neighbour 2 graph used to construct spatial weighting matrices for spatial analyses. Insets show permutation tests of sets of smoothed scalograms for the broad (1) and the fine (2) scales. The 95% quantile of the simulated *R^2^* distribution is indicated with crosses connected by a dotted line and significant *p*-values indicated on graph. Eigenvalue scores (black and whites squares) of the ordination with highest *R^2^* given in each case.
